# P01-016 – Decreased vitamin D levels in FMF patients

**DOI:** 10.1186/1546-0096-11-S1-A20

**Published:** 2013-11-08

**Authors:** H Onur, H Aral, V Arıca, G Bercem, M Usta, Ö Kasapçopur

**Affiliations:** 1 Department of Pediatrics, Istanbul Training and Research Hospital, Istanbul, Turkey; 2 Department of Biochemistry, Istanbul Training and Research Hospital, Istanbul, Turkey; 3 Department of Pediatrics, Mustafa Kemal University Medical Faculty, Hatay, Turkey; 4 Department of Biochemistry, Giresun University Medical Faculty, Giresun, Turkey; 5 Pediatric Rheumatology, Istanbul University, Cerrahpasa Medical Faculty, Istanbul, Turkey

## Introduction

Familial Mediterranean fever (FMF) patients suffer from recurrent self-limited inflammatory febrile attacks, abdominal, chest or joint pain. It is still unknown what triggers or ends these periodical attacks. Vitamin D, in addition phospho-calcium metabolism, has immunomodulation effects as pleotropic hormone. Vitamin D status has been linked to the occurrence and severity of autoimmune and inflammatory diseases.

## Objectives

The aim of this study was to determine whether vitamin D deficiency is present in patients with Familial Mediterranean fever (FMF) compared with healthy child individuals.

## Methods

The study group was comprised of 126 patients diagnosed with FMF (female/male (n):66/60); and 50 healthy control (female/male(n):25/25).Serum baseline 25-hydroxy vitamin D levels were measured. The FMF patients has divided into four groups according to mutation analysis.

## Results

Vitamin D levels in FMF patients and healthy controls were 24,47±8,48 and 28,70±11,70 ng/ml respectively. FMF patients had significantly decreased vitamin D levels compared with healthy controls (p<0,01). The study has shown plasma vitamin D level was similar in FMF patients with different MEFV gene mutation groups (P>0.05).The groups has been comprised as M694V/M694V(n=26), M694V/Other(n=38), Other/Other(n=46),Negative(n=16). The increase in age was significantly correlated with the decrease in vitamin D levels (r:-0.327 p<0.0001). Plasma vitamin D levels has not shown significance between patients with joint symptom(n:62) and without joint symptom (n:64) and has been detected as 23.72 ± 7.93, 25.20 ± 8.99 ng/ml respectively.(p =0.328).

## Conclusion

The etiology of recurrent attacks of serositis in familial Mediterranean fever (FMF) is not completely understood. Uncontrolled clinical case series have reported that factors associated with emotional, physiological, or physical stress precede and might trigger the attacks. In conclusion it is thought that vitamin D deficiency in pediatric FMF patients may have provide basis the attacks. Vitamin D level should be carefully examined and nutritional supplementation should be needed in FMF patients. Further studies with larger patient populations need to hold to investigate the vitamin D deficiency in patients with FMF.

## Disclosure of interest

None declared.

